# Effects of *Inonotus obliquus* Polysaccharides on Proliferation, Invasion, Migration, and Apoptosis of Osteosarcoma Cells

**DOI:** 10.1155/2020/4282036

**Published:** 2020-11-17

**Authors:** Baohui Su, Xuezhi Yan, Yuezhong Li, Junshan Zhang, Xiaoyan Xia

**Affiliations:** ^1^Department of Spinal Surgery, Weifang People's Hospital, Weifang, Shandong 261041, China; ^2^Department of Surgery, Anqiu Municipal Hospital, Weifang, Shandong 262100, China

## Abstract

**Objectives:**

To observe the effect of *Inonotus obliquus* polysaccharide (IOP) on the proliferation, invasion, migration, and apoptosis of osteosarcoma cells and to elucidate its underlying molecular mechanism.

**Methods:**

IOP was extracted from *Inonotus obliquus*, human osteosarcoma MG-63 cells and U2OS cells were cultured in vitro, and the effects of IOP on the proliferation, migration, invasion, and apoptosis of MG-63 cells and U2OS cells were determined by CCK-8 assays, cell scratch assays, transwell assays, and flow cytometry, respectively. Western blot was used to detect the expression of related proteins in the Akt/mTOR and NF-*κ*B signaling pathways.

**Results:**

Compared with the control group, MG-63 cells and U2OS cells treated with IOP of 80 *μ*g/ml, 160 *μ*g/ml, and 320 *μ* g/ml in the experimental group had significantly lower proliferation activity, decreased migration and invasion ability, and increased apoptosis rate (*P* < 0.05). Furthermore, IOP could significantly inhibit the activation of the Akt/mTOR and NF-*κ*B signaling pathway (*P* < 0.05).

**Conclusion:**

IOP can regulate the proliferation, migration, invasion, and apoptosis of osteosarcoma cells by inhibiting the activation of the Akt/mTOR signaling pathway. It has antitumor activity on osteosarcoma and has the potential of clinical application in osteosarcoma treatment.

## 1. Introduction

Osteosarcoma (OS) is a common malignant bone tumor that occurs mainly in adolescents and young people [1]. Osteosarcoma is an extremely aggressive malignant tumor characterized by rapid growth and early metastasis [2]. It is suggested that clinical prognosis from conventional osteosarcoma treatments such as surgery, radiotherapy, and chemotherapy is still not satisfactory [3]. Although new drugs have been applied in osteosarcoma chemotherapy, however, it could also result in strong drug resistance and unnecessary side effects, which can further cause serious problems [4]. Therefore, looking for a new targeted therapeutic drug is of great significance for preventing the progression of osteosarcoma and improving the survival of patients.

With the discovery of paclitaxel, the development of new tumor drugs has refocused on natural products. Particularly, in recent years, the application of natural products of medicinal plants in modern medicine is one of the important aspects of tumor treatment [5]. Among these natural products, polysaccharides are a class of biological macromolecules with great structural diversity, have no toxic effects, and have therapeutic values, so they have attracted widespread attention in the medical community [6]. Natural polysaccharides have a variety of pharmacological activities such as antitumor, immune regulation, antioxidation, antibacterial, and antiulcer [7]. Among them, their antitumor effects have an important application value. For example, many antitumor polysaccharides have been found to be nontoxic to normal cells and can induce apoptosis to various cancer cells [8, 9].

*Inonotus obliquus* fungus is a precious edible and medicinal fungus and has a long history as a traditional medicinal plant in China and other countries. It is reported that *Inonotus obliquus* has many biological functions, including antioxidant, antimitotic, and anti-inflammatory activities [10, 11]. In particular, *Inonotus obliquus* polysaccharide (IOP) extracted from *Inonotus obliquus* fungus can be used to treat and prevent various diseases such as cancer [12], diabetes [13], pancreatitis [14], high lipidemia [10], colitis [15], and Alzheimer's disease [16]. However, the effect of IOP on osteosarcoma and its possible mechanism are still poorly understood. This study is to investigate the effect of IOP on the proliferation, invasion, migration, and apoptosis of osteosarcoma cells and its associated molecular mechanism, so as to provide a basis for the application of IOP in the treatment of osteosarcoma.

## 2. Materials and Methods

### 2.1. Materials and Reagents

The CCK8 kit was purchased from MedChemExpress in the United States, the transwell cell was purchased from Corning in the United States, and the Annexin V-FITC/PI apoptosis kit was purchased from Sigma-Aldrich in the United States. Phospho-Akt (Ser473) antibody, Phospho-Akt (Thr308) antibody, Akt (pan) antibody, mTOR antibody, and Phospho-mTOR (Ser2448) antibody were purchased from Cell Signaling Technology, USA.

### 2.2. IOP Extraction and Identification

IOP was extracted according to the method of Xu et al. [17]. First, use the savage method to dissolve and remove the protein of Inonotus obliquus, then dialysis with tap water for 24 hours, and then with distilled water for 12 hours. The crude polysaccharide was centrifuged, lyophilized, and further purified in an anion exchange DEAE cellulose column (50cm × 2.6cm). Elute with 0 M, 0.25 m, and 0.5 m NaCl solution at a flow rate of 0.4 ml/min. Elute with 0.15 m NaCl solution in a Sephadex G-200 gel column (1.6cm × 40cm) at a flow rate of 0.18 ml/min. Collect the IOP, make it into a lyophilized powder, and store in an airtight container at 4°C.

### 2.3. Cell Culture and Treatment

Human osteosarcoma MG-63 cells and U2OS cells were purchased from Shanghai Fuheng Biotechnology Co., Ltd. and cultured in DMEM high-sugar medium containing 10% fetal bovine serum under 37°C with 5% CO_2_. 10 *μ*g/mL, 20 *μ*g/mL, 40 *μ*g/mL, 80 *μ*g/mL, 160 *μ*g/mL, and 320 *μ*g/mL of IOP were added to the medium for the treatments, and a group of cells without IOP was used as a negative control (NC).

### 2.4. Cell Proliferation Detected with CCK-8

MG-63 cells and U2OS cells were seeded in 96-well plates and treated with different concentrations of IOP. After the cells were incubated in the incubator for 24, 48, and 72 h, 10 *μ*l of CCK8 solution was added to each well and incubated for a while. The absorbance at 450 nm was measured with a spectrophotometer afterward.

### 2.5. Cell Migration Measured with Cell Scratch Test

Cell migration experiments were carried out using scratch experiments. Specifically, MG-63 cells and U2OS cells were seeded on 6-well plates, cultured overnight, and then treated with different concentrations of IOP for 24 h. After that, scratch the cell layer with a sterile plastic pipette tip to form a scratch. The detached cells were rinsed off with PBS. After that, the DMEM medium containing 1% fetal bovine serum was added, the cells were cultured for 24 h, photos were taken, and the cell migration rate was calculated accordingly.

### 2.6. Cell Invasion Determined by Transwell Cell Assay

Cell invasion tests were carried out using a matrix gel-coated transwell cell. Specifically, 1.0 × 10^5^ MG-63 cells or U2OS cells treated with different concentrations of IOP for 24 h were inoculated into the upper chamber with 100 ml of serum-free DMEM, and 500 ml of medium containing 10% FBS was added to the lower chamber. After 24 h of incubation, the cells on the surface of the upper membrane were wiped with a cotton swab, and the cells passing through the membrane were fixed with methanol, stained with crystal violet, and counted afterward.

### 2.7. Cell Apoptosis Measured with Annexin V-FITC/PI Apoptosis Detection Kit

MG-63 cells and U2OS cells were seeded in 6-well plates at 4 × 10^5^ cells per well and cultured overnight. The cells were treated with different concentrations of IOP for 24 h. Cells were then collected, washed with PBS, and double-stained using Annexin V-FITC/PI apoptosis detection kit. Finally, flow cytometry was used to analyze the cell apoptotic rate.

### 2.8. Western Blot

After the cells were treated with different concentrations of IOP for 24 h, the cells were washed twice with cold PBS. Total protein was then extracted using a RIPA lysis buffer supplemented with a protease inhibitor, and total protein was then quantified using a BCA protein concentration determination kit. An equal amount of protein (20 *μ*g/well) from each treatment was taken and subjected to SDS-PAGE electrophoresis, and the protein was then electrotransferred to a PVDF membrane. The membrane was then blocked with 5% skim milk at room temperature for 1 h and then incubated with the primary antibody overnight at 4°C. The membrane was then washed and was incubated with the secondary antibody for 1 h at room temperature. Protein bands were then detected using the ECL luminescence kit.

### 2.9. Statistical Analysis

All the data in this study were analyzed using SPSS software. Results are expressed as mean ± standarddeviation. The *t*-test and one-way analysis of variance was used to compare the differences between groups. The difference was statistically significant at *P* < 0.05.

## 3. Results

### 3.1. IOP Identification by High-Performance Liquid Chromatography

**Figure 1:**
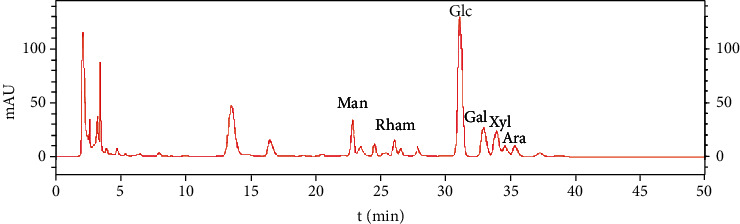
HPLC chart of IOP.

IOP was analyzed with high-performance liquid chromatography as shown in Figure 1. We analyzed the monosaccharide composition of IOP. The monosaccharide composition of IOP is Man, Rha, Glu, Gal, Xyl, and Ara, and the molar ratio is 2.2 : 1.1 : 11.8 : 2.8 : 2.7 : 1.0.

### 3.2. IOP Inhibits MG-63 Cells and U2OS Cell Proliferation

**Figure 2:**
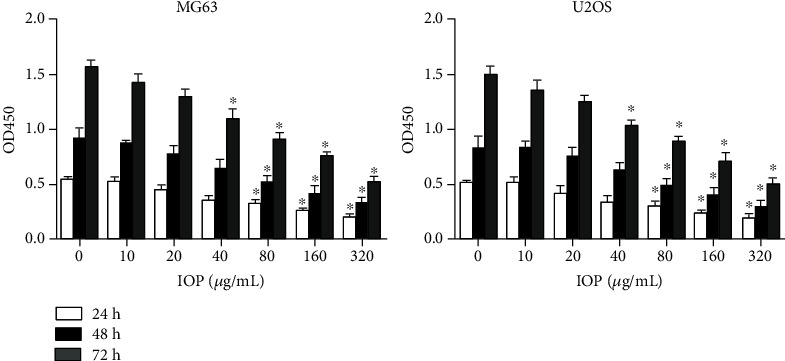
The effect of IOP on the proliferation activity of MG-63 cells and U2OS cells evaluated with CCK8. ^∗^*P* < 0.05.

As shown in Figure 2, CCK8 assay was used to measure the proliferation of MG-63 cells and U2OS cells, and after IOP was added to MG-63 cells and U2OS cells at different concentrations (10 *μ*g/mL, 20 *μ*g/mL, 40 *μ*g/mL, 80 *μ*g/mL, 160 *μ*g/mL, and 320 *μ*g/mL), compared with the control group, the cell proliferation activity was significantly reduced (*P* < 0.05) when the IOP was at the concentrations of 80 *μ*g/mL, 160 *μ*g/mL, and 320 *μ*g/mL after 48 h and 72 h of treatments. This result indicates that IOP can inhibit the proliferation of osteosarcoma cells.

### 3.3. IOP Inhibits MG-63 Cells and U2OS Cell Migration

**Figure 3:**
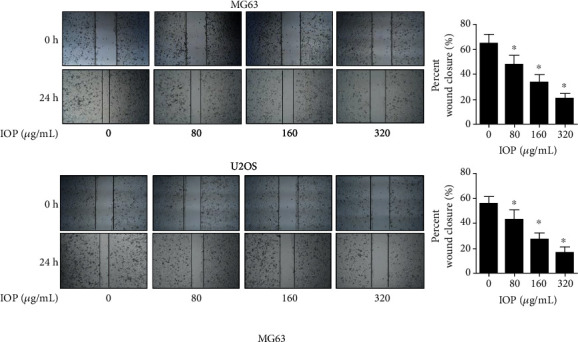
The effect of IOP on the migration activity of MG-63 cells and U2OS cells evaluated with the cell scratch test. ^∗^*P* < 0.05.

As shown in Figure 3, after adding IOP to MG-63 cells and U2OS cells at different concentrations (80 *μ*g/mL, 160 *μ*g/mL, and 320 *μ*g/mL) compared with the control group, the migration activity of the cells was significantly reduced in the 80 *μ*g/mL, 160 *μ*g/mL, and 320 *μ*g/mL groups (*P* < 0.05). It is apparent that this inhibition on the MG-63 cells and U2OS cell migration by the treatment of IOP was in a dose-dependent manner. Overall, this result indicates that IOP can inhibit the migration of osteosarcoma cells.

### 3.4. IOP Inhibits MG-63 Cells and U2OS Cell Invasion

**Figure 4:**
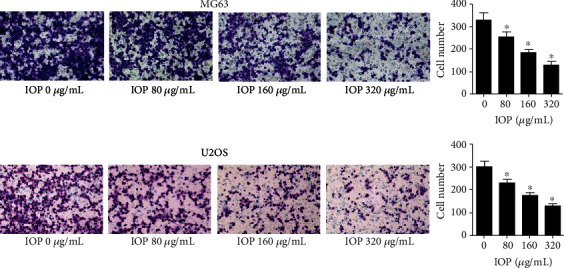
The effect of IOP on the invasion ability of MG-63 cells and U2OS cells was determined with the transwell experiment. ^∗^*P* < 0.05.

As shown in Figure 4, when IOP was added to MG-63 cells and U2OS cells at different concentrations (80 *μ*g/mL, 160 *μ*g/mL, and 320 *μ*g/mL) compared with the control group, the invasive ability of the cells was significantly reduced in the 80 *μ*g/mL, 160 *μ*g/mL, and 320 *μ*g/mL groups (*P* < 0.05). And this inhibition of cell invasion by the treatment of IOP was in a concentration-dependent manner. This result indicates that IOP can inhibit the invasion of osteosarcoma cells.

### 3.5. IOP Promotes Apoptosis of MG-63 Cells and U2OS Cells

**Figure 5:**
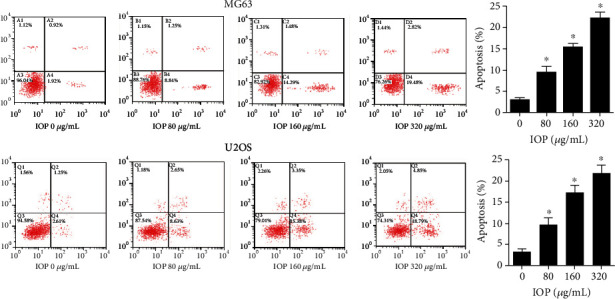
The effect of IOP on the apoptosis of MG-63 cells and U2OS cells was measured with flow cytometry. ^∗^*P* < 0.05.

As shown in Figure 5, after adding IOP to MG-63 cells and U2OS cells at different concentrations (80 *μ*g/mL, 160 *μ*g/mL, and 320 *μ*g/mL), compared with the control group, the apoptosis rate of the cells was significantly increased in the 80 *μ*g/mL, 160 *μ*g/mL, and 320 *μ*g/mL groups (*P* < 0.05), which was also in a dose-dependent manner. This result indicates that IOP can promote the apoptosis of osteosarcoma cells.

### 3.6. IOP Inhibits Akt/mTOR and NF-*κ*B Signaling Pathway

**Figure 6:**
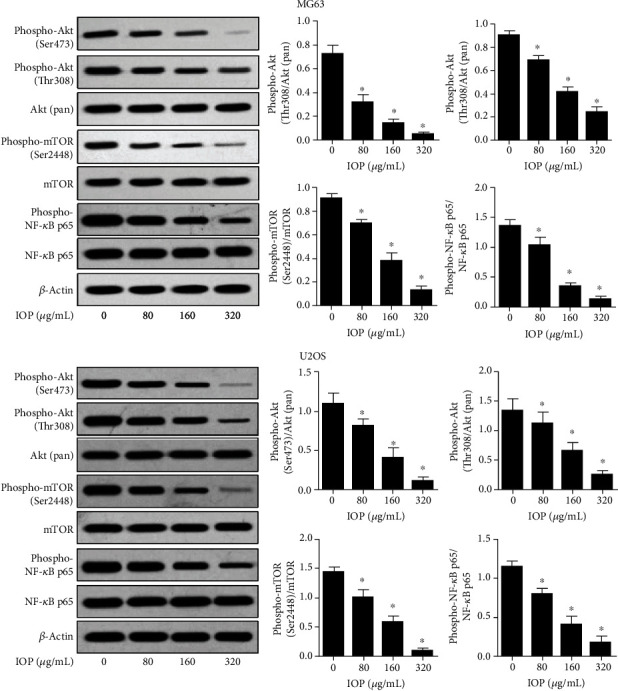
Effect of IOP on Akt/mTOR and NF-*κ*B signaling pathway-related protein expression detected with Western blot. ^∗^*P* < 0.05.

As shown in Figure 6, after adding IOP to MG-63 cells and U2OS cells at different concentrations (80 *μ*g/mL, 160 *μ*g/mL, and 320 *μ*g/mL), compared with the control group, the phosphorylation levels of Akt and mTOR as well as NF-*κ*B p65 were significantly reduced (*P* < 0.05) at all treatment groups. And with the increase of the IOP levels, the phosphorylation of Akt at Ser473 and Thr308 as well as the phosphorylation of mTOR at Ser2448 and NF-*κ*B p65 decreased dramatically. This result indicates that IOP can inhibit the activation of the Akt/mTOR and NF-*κ*B signaling pathways.

## 4. Discussion

Osteosarcoma is one of the most common malignant bone tumors, which seriously endangers the health of adolescents. In recent years, with the development of chemotherapy drugs, the survival rate of osteosarcoma has improved; however, the overall effectiveness is still not satisfactory. Drug resistance and side effects are the leading causes of cancer treatment failure. Therefore, it is imperative to seek alternative methods, and it has become a hot spot for research. In China, IOP has been used as a folk medicine for treating cancer and other diseases for thousands of years [18]. However, little is known about the mechanisms behind it. Previous studies have shown that IOP can change energy metabolism through the LKB1/AMPK pathway and induce lung cancer cell apoptosis [18]; IOP can also reduce the expression of MMP-2 and MMP-9 by downregulating the NF-*κ*B signaling pathway, thereby inhibiting the migration and invasion of B16-F10 cells [19]. This study intended to elucidate the role of IOP in osteosarcoma and its mechanism of inhibiting osteosarcoma cells and explore its potential value as a chemotherapeutic agent for osteosarcoma.

In this study, we first isolated and purified the polysaccharide IOP from Inonotus obliquus fungus and investigated its effect on the proliferation, migration, invasion, and apoptosis of osteosarcoma MG-63 cells and U2OS cells in vitro. CCK-8 experiments showed that IOP significantly inhibited the proliferation of MG-63 cells and U2OS cells. In particular, when the concentration of IOP was greater than 80 /mL, the proliferation activity of MG-63 cells and U2OS cells was significantly downregulated compared with the control group. Similarly, cell scratch experiments have confirmed that IOP has a significant inhibitory effect on the migration capacity of MG-63 cells and U2OS cells, transwell experiments have shown that IOP has a significant inhibitory effect on the invasiveness of MG-63 cells and U2OS cells, and further apoptosis experiments have demonstrated that IOP can promote the apoptosis of MG-63 cells and U2OS cells. Therefore, taken all these together, our functional experiments showed that IOP has obvious antitumor activity on osteosarcoma cells and has the potential for clinical application in osteosarcoma treatment.

Further studies suggested that IOP can also inhibit the activation of the Akt/mTOR and NF-*κ*B signaling pathway in osteosarcoma cells. Akt, also known as PKB (protein kinase B), is a serine/threonine-specific protein kinase that can be activated by PI3K at Thr308 and Ser473 sites. Activated Akt can activate the mTOR pathway in a variety of ways. And mTOR is an important serine-threonine protein kinase downstream of the PI3K/Akt signaling pathway which can regulate the malignant biological process of tumor cells by activating ribosomal kinase [20]. The Akt/mTOR signaling pathway is typically abnormally activated in a variety of malignant tumors and causes abnormal expression of downstream genes such as PFKFB2, p21, Vimentin, and Bcl-2.

Thereby, Akt/mTOR signaling pathway plays an important role in a variety of cellular biological processes of tumor cells, including autophagy, proliferation, migration, invasion, and apoptosis [21]. NF-*κ*B has been shown to control the expression of tumor-related genes, such as Cyclin D1, c-Myc, HER2, interleukins, and XIAP, to regulate chronic inflammation, tumor cell survival, antiapoptosis, proliferation, invasion, and angiogenesis [22]. RelA/p65, which is a subunit of NF-*κ*B, can increase transcription activity after phosphorylation, causing the activation of NF-*κ*B, thereby promoting the transcription of related oncogenes [23]. In this study, it appears that IOP may regulate the proliferation, migration, invasion, and apoptosis of osteosarcoma cells by inhibiting the activation of the Akt/mTOR and NF-*κ*B signaling pathway and further supplement the understanding of the antitumor mechanism of IOP.

## 5. Conclusion

The results of this study indicate that polysaccharide from *Inonotus obliquus* polysaccharide can regulate the proliferation, migration, invasion, and apoptosis of osteosarcoma cells by inhibiting the activation of the Akt/mTOR and NF-*κ*B signaling pathway. This study also confirms that IOP has obvious antitumor activity against osteosarcoma and has the potential for clinical application in osteosarcoma treatment. At the same time, the molecular study provides a theoretical basis for the application of IOP in the treatment of osteosarcoma. However, this study lacks in vivo animal and clinical experiments, which are the shortcomings of this study and the direction for future development.

## Data Availability

All the data is available with the handwritten notebook documented in our lab.
